# Origins of the Greenland shark (*Somniosus microcephalus*): Impacts of ice‐olation and introgression

**DOI:** 10.1002/ece3.3325

**Published:** 2017-09-08

**Authors:** Ryan P. Walter, Denis Roy, Nigel E. Hussey, Björn Stelbrink, Kit M. Kovacs, Christian Lydersen, Bailey C. McMeans, Jörundur Svavarsson, Steven T. Kessel, Sebastián Biton Porsmoguer, Sharon Wildes, Cindy A. Tribuzio, Steven E. Campana, Stephen D. Petersen, R. Dean Grubbs, Daniel D. Heath, Kevin J. Hedges, Aaron T. Fisk

**Affiliations:** ^1^ Department of Biological Science California State University Fullerton CA USA; ^2^ Great Lakes Institute for Environmental Research University of Windsor Windsor ON Canada; ^3^ Department of Natural Resources and the Environment Wildlife and Fisheries Conservation Center and Center for Environmental Sciences and Engineering University of Connecticut Storrs CT USA; ^4^ Biological Sciences University of Windsor Windsor ON Canada; ^5^ Justus Liebig University Giessen Germany; ^6^ Fram Centre Norwegian Polar Institute Tromsø Norway; ^7^ Department of Biology University of Toronto Mississauga Mississauga ON Canada; ^8^ Faculty of Life and Environmental Sciences University of Iceland Reykjavík Iceland; ^9^ Department of Fisheries and Wildlife Michigan State University East Lansing MI USA; ^10^ Mediterranean Institute of Oceanography (MIO) UM 110 Aix‐Marseille University CNRS/INSU Toulon University IRD Marseille France; ^11^ Auke Bay Laboratories AFSC/NMFS/NOAA/DOC Ted Stevens Marine Research Institute Juneau AK USA; ^12^ Conservation and Research Department Assiniboine Park Zoo Winnipeg MB Canada; ^13^ Coastal and Marine Laboratory Florida State University St. Teresa FL USA; ^14^ Arctic Aquatic Research Division Fisheries and Oceans Canada Winnipeg MB Canada

**Keywords:** elasmobranch, Greenland shark, interspecific gene flow, introgressive hybridization, isolation with migration, *Somniosus microcephalus*

## Abstract

Herein, we use genetic data from 277 sleeper sharks to perform coalescent‐based modeling to test the hypothesis of early Quaternary emergence of the Greenland shark (*Somniosus microcephalus*) from ancestral sleeper sharks in the Canadian Arctic‐Subarctic region. Our results show that morphologically cryptic somniosids *S. microcephalus* and *Somniosus pacificus* can be genetically distinguished using combined mitochondrial and nuclear DNA markers. Our data confirm the presence of genetically admixed individuals in the Canadian Arctic and sub‐Arctic, and temperate Eastern Atlantic regions, suggesting introgressive hybridization upon secondary contact following the initial species divergence. Conservative substitution rates fitted to an Isolation with Migration (IM) model indicate a likely species divergence time of 2.34 Ma, using the mitochondrial sequence DNA, which in conjunction with the geographic distribution of admixtures and Pacific signatures likely indicates speciation associated with processes other than the closing of the Isthmus of Panama. This time span coincides with further planetary cooling in the early Quaternary period followed by the onset of oscillating glacial‐interglacial cycles. We propose that the initial *S. microcephalus*–*S. pacificus* split, and subsequent hybridization events, were likely associated with the onset of Pleistocene glacial oscillations, whereby fluctuating sea levels constrained connectivity among Arctic oceanic basins, Arctic marginal seas, and the North Atlantic Ocean. Our data demonstrates support for the evolutionary consequences of oscillatory vicariance via transient oceanic isolation with subsequent secondary contact associated with fluctuating sea levels throughout the Quaternary period—which may serve as a model for the origins of Arctic marine fauna on a broad taxonomic scale.

## 1 INTRODUCTION

For highly mobile marine organisms with extensive distributions, the mechanisms driving genetic divergence and speciation can be difficult to discern. The lack of obvious physical barriers to gene flow in the marine environment offers the potential for genetic connectivity over broad geographic and temporal scales with some species showing little genetic differentiation even among different oceanic basins (e.g., sharks and bony fishes: Schmidt et al., 2009; Roy, Hardie, Treble, Reist, & Ruzzante, 2014; da Silva Ferrette et al., 2015). Classically, marine speciation was largely thought to occur in allopatry through vicariance (Avise, 1992; Palumbi, 1994), whereby geologic isolating barriers (i.e., mountain chains, isthmuses) or hydrologic phenomena (oceans, rivers, drainage basins, and sea ice cover) isolate formerly large species distributions (Briggs, 2006; Corrigan & Beheregaray, 2009; Gaither et al., 2010; Knowlton & Weigt, 1998; O'Corry‐Crowe et al., 2016). Numerous challenges to the notion of allopatry as the primary driver of marine divergence exist (Rapoport, 1994; Kinlan & Gaines, 2003; Palumbi, 2004; in Norris & Hull, 2012), suggesting that constraints to gene flow and/or strong selection can also promote genetic differentiation among marine populations in the absence of physical barriers (Coyne & Orr, 2004; Horne, van Herwerden, Choat, & Robertson, 2008; Miglietta, Faucci, & Santini, 2011; Palumbi, 1994). Marine examples of “soft vicariance” (e.g., isolation in oceanic currents; see Hickerson & Meyer, 2008; Cowman & Bellwood, 2013), isolation at larger regional spatial scales (Mach et al., 2011), drift effects along environmental gradients (Ingram, 2011; Roy, Hurlbut, & Ruzzante, 2012), local adaptation (Bremer, Mejuto, Greig, & Ely, 1996; Clarke, Munch, Thorrold, & Conover, 2010), and ecological diversification in sympatry (Crow, Munehara, & Bernardi, 2010; Puebla, 2009; Rocha, Robertson, Roman, & Bowen, 2005), have all been documented in marine taxa with the potential for widespread connectivity.

In Arctic marine environments, the evolutionary histories and population demographics of numerous endemic species appear to be influenced strongly by fluctuations in the extent of sea ice associated with cooling glacial and warming interglacial cycles since the onset of the geologic Quaternary period roughly 2.6 Ma ago (e.g., O'Corry‐Crowe et al., 2010). Genetic structure in beluga whales *Delphinapterus leucas* (O'Corry‐Crowe, Suydam, Rosenberg, Frost, & Dizon, 1997; O'Corry‐Crowe et al., 2010) and walruses *Odobenus rosmarus* (Andersen & Born, 2000) provides strong indicators that ice effects during the Pleistocene (0.0117–2.588 Ma ago), coupled with additional ecological factors such as large‐scale temporal variations in levels of primary productivity, can leave lasting genetic imprints in a species' evolutionary history. Range expansions and contractions have also been shown in other Arctic and sub‐Arctic species (Andersen et al., 1998; O'Corry‐Crowe, 2008; O'Corry‐Crowe et al., 1997; O'Corry‐Crowe et al., 2010; Palsbøll, Heide‐Jorgensen, & Dietz, 1997; Palo, 2003), associated with sequential cooling and warming periods, respectively. Specifically, for beluga whales, late Pleistocene oscillations (7–36 ka) in sea ice extent may explain recent population subdivisions, with genetic support for recurrent episodes of gene flow that likely coincided with warming periods (O'Corry‐Crowe et al., 2010). Species divergence, occurring with such episodic gene flow, can have important impacts on population demographic parameters such as connectivity and size (Hey, 2010b; Moore, Gow, Taylor, & Hendry, 2007; Nosil, 2008), which can then lead to substantial alteration of a species' adaptive potential (Eizaguirre & Baltazar‐Soares, 2009; Hellberg, 2009; Pelletier, Garant, & Hendry, 2009). Estimating these parameters is, therefore, an important step toward a better understanding of the evolutionary histories and potential trajectories of different species, especially considering the dynamic environmental conditions that likely govern potential gene flow among them (Seehausen et al., 2008). Disentangling the interplay among demographic parameters, environmental fluctuations, and/or the potential for gene flow, however, can be especially complicated among closely related cryptic sister species whose taxonomic differentiation remains nebulous (Griffiths et al., 2010). Such information is nevertheless urgently needed, especially for species whose main distribution includes large Arctic marine ecosystems, which are currently experiencing major climate‐related transformations (Wassmann, Duarte, Agusti, & Sejr, 2011).

The number of currently recognized *Somniosus* species varies according to sources, with species regarded as geographically isolated from one another despite little differences in physical appearance having occurred among sister taxa over their evolutionary histories (Yano, Stevens, & Compagno, 2004). Greenland sharks (*Somniosus microcephalus*) are known from the Canadian Arctic eastward, including Greenland and Svalbard through to the Laptev Sea (Chernova et al. 2015), south to Iceland and Nova Scotia on the eastern seaboard of Canada (MacNeil et al., 2012) in the western Atlantic and south to the United Kingdom and southernmost Norway (in deepwater areas). Pacific sleeper sharks (*Somniosus pacificus*), on the other hand, range from the Bering Sea south throughout the deep waters of the Pacific and into the Southern Ocean (Yano et al., 2004). The smaller *Somniosus rostratus* is reported from the Mediterranean Sea and the Northeastern temperate Atlantic Ocean, while a fourth species, *Somniosus antarcticus* has been described from the Southern Ocean (Yano et al., 2004). Current species delimitations, notwithstanding accurate species identification and population delineation for the larger somniosids, (*S. microcephlaus*,* S. pacificus,* and *S. antarcticus*) remain challenging given their overall morphological similarities and the fact that even the few “distinguishing” characteristics for each species often considerably overlap (Benz et al., 2007; MacNeil et al., 2012).

Using partial mitochondrial (mtDNA) cytochrome *b* (cyt *b*) gene sequences, Murray et al. (2008) demonstrated that Pacific sleeper sharks and Greenland sharks can be distinguished from one another, with the former exhibiting no geographic structure among samples collected across the Pacific Ocean or more southerly latitudes. More recently, genetic characterization of juvenile Greenland sharks from the Canadian Arctic revealed individuals carrying Pacific sleeper shark mtDNA haplotypes (Hussey et al., 2015). This finding, taken with a growing list of cytonuclear discordance and/or genetic admixture among closely related elasmobranchs in the literature, suggests that hybridization among *Somniosus* species is possible (Hinojosa‐Alvarez, Walter, Diaz‐Jaimes, Galván‐Magaña, & Paig‐Tran, 2016; Kashiwagi, Marshall, Bennett, & Ovenden, 2012; Morgan et al., 2012; Walter et al., 2014). Questions remain whether this potential hybridization is recently developed among once more distinguishable species, whether it results from long‐term gene flow between sister species, or whether the shared genetic signatures are a product of incomplete lineage sorting (retained ancestral polymorphism).

In this work, we focus on genetic variation in the Greenland shark across its current known distribution. Our objective was to compare patterns of DNA sequence polymorphism and divergence within and among Greenland sharks sampled throughout the Arctic, and sub‐Arctic‐temperate regions, with those of the closely related Pacific sleeper shark, and other deepwater somniosids in order to develop a more comprehensive picture of the origins of the Greenland shark.

## 2 METHODS

### 2.1 Tissue samples

**Figure 1:**
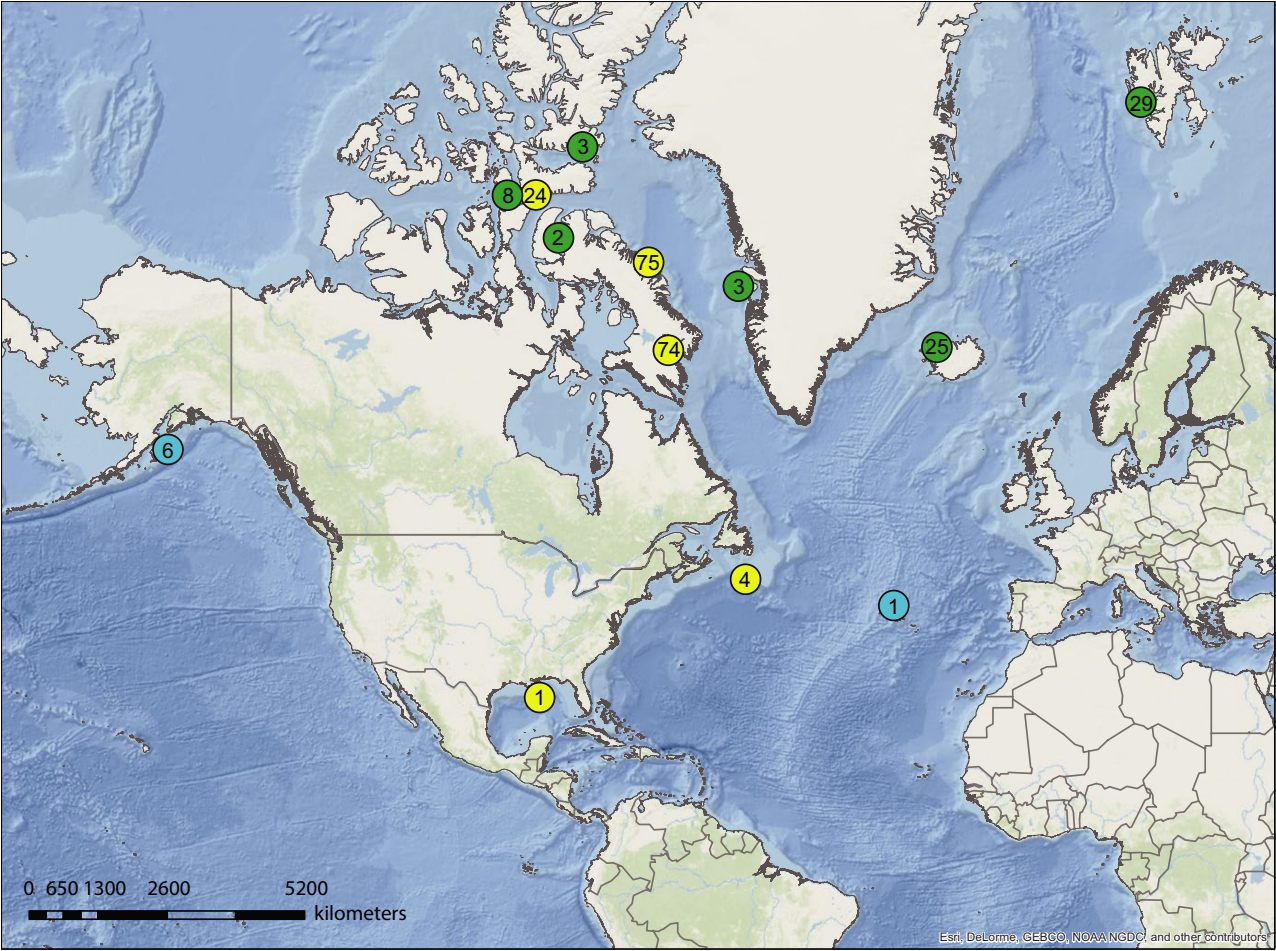
Sampling locations of somniosid sharks in the Arctic, North Atlantic Ocean, and North Pacific: *Somniosus microcephalus* (green); *Somniosus pacificus* (blue) and; cytonuclear discordant individuals (yellow). Numbers inside circles are sample sizes

Tissue samples from 247 somniosid sharks were collected across a wide distribution including the Canadian Arctic and sub‐Arctic, Western Greenland, Svalbard, Iceland, and the North Atlantic (Fig. 1) that were identified as Greenland shark (i.e., *S. microcephalus*) during sample collection. Pacific sleeper shark (*S. pacificus*;* n *=* *6) tissue samples were collected from Alaska. Additional putative *S. microcephalus* individuals (e.g., identified as *S. microcephalus* in the field) were collected from the Mid‐Atlantic Ridge (MAR‐Azores; *n *=* *1) and the Gulf of Mexico (GM; *n *=* *1), and published *Somniosus* mtDNA sequences (*n *=* *22) from Murray et al. (2008) were retrieved from GenBank (EF090943–EF090963) and included for comparative purposes (*n *=* *277 *Somniosus* spp. samples total).

### 2.2 Molecular methods

Genomic DNA was extracted from all samples using either the standard phenol–chloroform method or the Promega Wizard Extraction kit following the manufacturer's instructions (Promega Corp, Madison WI, USA). We partially sequenced the mtDNA cytochrome *b* (cyt *b*) gene, the nuclear recombination activation gene (RAG1), and the nuclear internal transcribed spacer 2 (ITS2) with the flanking 5.8 and 28 ribosomal sequences (5.8S and 28S) herein referred to as the ITS2 locus, for each sampled individual. PCR amplification and sequencing reaction conditions for the markers used are available in the Appendix S1.

### 2.3 Data analysis

All derived mtDNA sequences were aligned with published GenBank sequences in SEQUENCHER 5.0 (GeneCodes, Ann Arbor, MI) and trimmed to 702 bp. Nuclear sequences were also separately aligned in SEQUENCHER and trimmed to 659 bp and 1,085 bp for RAG1 and ITS2, respectively. To estimate genealogical relationships among haplotypes, a 95% statistical parsimony network was constructed in TCS 1.21 (Clement, Posada, & Crandall, 2000) for each locus. Locus‐specific nucleotide models were determined in jModelTest 2.1.10 based on *AIC/BIC* model comparisons (Darriba, Taboada, Doallo, & Posada, 2012). Haplotype diversity (Hd) and nucleotide diversity (π), Fu's *F* statistic, Tajima's *D,* genetic distance (*D*_a_), and tests for recent population expansion from mismatch distributions using cyt *b* sequences were performed in DnaSP 5.10.01 (Librado & Rozas, 2009). Estimates of genetic differentiation, *F*_ST_ (frequency‐based) and Φ_ST_ (Tamura‐Nei) among *S*. *microcephalus* sampling sites were calculated in Arlequin 3.5 (Excoffier, Laval, & Schneider, 2005) using the mtDNA data. A demographic history for *S. microcephalus* based on the cyt *b* dataset (using HKY+I model derived from jModelTest results) was reconstructed using the Bayesian Skyline Plot (BSP) model (Drummond, Rambaut, Shapiro, & Pybus, 2005) as implemented in the BEAST 1.8.0 package (Drummond, Suchard, Xie, & Rambaut, 2012). Four independent runs were performed, each using 100 mol/L generations and a sampling frequency of 5,000. Each run also used a piecewise‐constant skyline model, with 20 groups, and an uncorrelated lognormal relaxed‐clock mean rate (ucld.mean) was set to 0.0007 = 7 × 10^−10^ Ma^−1^ as per Martin, Naylor, and Palumbi (1992). Replicates were combined using LogCombiner (BEAST package), applying a burn‐in of 50% for each replicate. Finally, the BSP was visualized in the program Tracer 1.5 (Rambaut & Drummond, 2007) to detect changes in population size over time.

### 2.4 Coalescent analysis and divergence estimation

To estimate long‐term demographic parameters, namely *N*_e_, migration rates (*M*; i.e., measures of gene flow) and the time since divergence (*t*_0_) between the Greenland shark and the Pacific sleeper shark, we used Isolation with Migration (IM) models as implemented in IMa2 (Hey, 2007, 2010a). IM models search parameter space for the most likely genetically based genealogy describing the split between two taxonomic groups, using a Bayesian framework. Estimated genealogies are generated assuming random mating within groups and that neither group exchanges genetic materials with other nonsampled groups (Hey, 2010a; Hey & Nielsen, 2007). Generated genealogies specify branch lengths and group‐specific genetic variation (estimated as θ) from which demographic parameters can be computed. IM models were run using the mtDNA cyt *b* data, with the HKY model of sequence evolution. An initial hypothesized phylogeny between the two species was provided, and uniform priors were set regarding the distribution of each parameter of interest using preliminary runs. Three different substitution rates, each characterizing divergence among elasmobranchs: a conservative estimate (7 × 10^−10^ Ma^−1^; Martin et al., 1992), *Squalus* (9.90 × 10^−10^ Ma^−1^; Winchell, Martin, & Mallatt, 2004), and *Squatina* (1.67 × 10^−9^ Ma^−1^; Winchell et al., 2004) at cyt *b* were used to estimate cyt *b* substitution rates in this work. These substitution rates respectively range from very conservative (Martin & Palumbi, 1993; Martin et al., 1992) to those possibly more reflective of somniosids (e.g., rates derived for *Squalus* and *Squatina*), respectively. Demographic parameter searches were performed with 20 Markov chain Monte Carlo (MCMC) simulations using a geometric heating scheme to ensure adequate mixing among them. Initial searches were set to run between 1 x 10^6^ and 12 x 10^6^ iterations in order for the genealogies to reach a stationary distribution from which to sample. Once stationarity was reached, genealogies were permutated 10 x 10^6^ times and sampled every 100th step for a total of 100,000 recorded genealogies from which demographic parameters were calculated (see IMa2 manual; Hey, 2010a). The different searches were repeated three times, and all derived parameters were compared among all three iterations to evaluate consistency (Hey, 2010a). All genealogies recovered from each search were used to compare the information content and thus likelihood (L‐mode) of different IM models. To check for consistency in estimated parameters, model comparison with searches omitting some parameters was also run by specifically setting migration rate priors to zero, hereafter referred to as Isolation Only (ISO). ISO models prevent any migration rate influences on estimated demographic parameters and generate parameter estimates consistent with an incomplete lineage sorting (ILS) scenario.

Due to low levels of polymorphism at nuclear loci, IM analyses were not performed for the RAG1 and ITS2 datasets. Nuclear nucleotide substitution rates for RAG1 (4.85 × 10^−10^ Ma^−1^; Eytan & Hellberg, 2010) and ITS2 (1.50 × 10^−9^ Ma^−1^; Montoya‐Burgos, 2003) were also used to estimate RAG1 and ITS2 divergence times using a simple formula whereby the number of observed mutations at a locus is divided by the estimated gene‐wide percent divergence rate per Ma.

## 3 RESULTS

**Figure 2:**
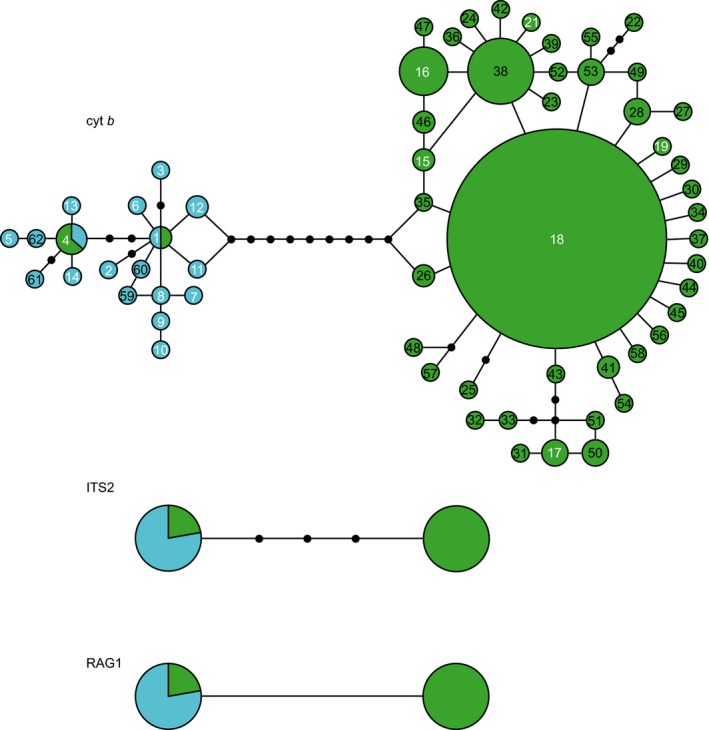
Haplotype variation at cyt *b*, ITS2, and RAG1 loci among sampled somniosids as depicted through a 95% statistically parsimony network. For cyt *b,* the size of the circles is proportional to the frequency of each haplotype in the 277 individual dataset, ITS2 and RAG1 haplotypes are not scaled for frequency. Haplotype numbers correspond to Table S1, white numbers are previously reported haplotypes, black numbers are novel haplotypes to this study. Individuals identified in the field as *Somniosus microcephalus* possess haplotypes in green, while those identified as *Somniosus pacificus* possess blue haplotypes

A total of 61 mtDNA haplotypes were recovered across all sleeper shark samples forming two distinct and well‐differentiated mtDNA haplogroups (Fig. 2, Tables S1 and S2). The Greenland shark samples consisted of 45 haplotypes, including 30 that are novel to this study (green circles with black text Fig. 2; see Table S2). These samples showed a haplotype diversity (Hd) of 0.7100 and nucleotide diversity (π) of 0.0028, while those recovered for the Pacific sleeper sharks were 0.9700 and 0.0053, for Hd and π, respectively (Table S2). A total of 18 haplotypes were among the Pacific sleeper shark samples, including four novel to this study. Site‐by‐site statistics are reported in Table 1. DNA sequences are deposited in GenBank under the accession numbers MF537353‐MF537393 and MF555591‐MF555594.

**Table 1:** Description of collection and mitochondrial sequence genetics‐based data for Somniosids included in the present study. *n*, sample size; *S*, number of polymorphic sites; *h*, number of haplotypes; Hd, haplotype diversity; *k*, average number of sequence pairwise differences; π, nucleotide diversity. Sample sites are AK, Alaska; CS, Cumberland Sound; DI, Disko Island; GF, Grice Fjord; IC, Iceland; KP, Kakiak Point; MB, Maxwell Bay; NS, Nova Scotia; RB, Resolute Bay; SI, Scott Inlet; SV, Svalbard; GM, Gulf of Mexico; MA, Mid‐Atlantic Ridge; GB, GenBank

Site	*n*	*S*	*h*	Hd	*k*	π	Admixtures
AK	6	8	5	0.933	3.467	0.00494	0
CS	74	33	20	0.805	2.422	0.00345	2
DI	3	6	3	1.000	4	0.00570	0
GF	3	2	3	1.000	1.333	0.00190	0
IC	25	10	10	0.720	1.2	0.00171	0
KP	2	2	2	1.000	2	0.00285	0
MB	24	21	6	0.543	2.032	0.00290	1
NS	4	14	3	0.833	7.167	0.01021	1
RB	8	11	6	0.893	30.36	0.00432	0
SI	75	27	17	0.764	2.282	0.00883	3
SV	29	11	10	0.695	1.596	0.00227	0
GM	1	—	1	—	—	—	1
MA	1	—	1	—	—	—	—
GB	22						

Only two alleles were recovered from each of the nuclear markers, with these alleles characterized by mostly species‐specific single nucleotide polymorphisms (SNPs). For the RAG1 marker, a “C” was predominant at position 607 in identified Pacific sleeper sharks, while a “T” was prevalent in Greenland sharks. Four polymorphic positions were found in 1,085 bases of the ITS2 sequences, a single indel and three SNPs. Greenland sharks possessed an “A” insertion at position 67. For the SNPs, Greenland sharks displayed an “A” at position 72, a “G” at position 142, and an “A” at position 507; while Pacific sleeper sharks possessed a “G” at position 72, a “C” at position 142, and a “C” at position 507.

Some sampled individuals reported as Greenland sharks in the field, and subsequently genotyped, showed a mixture of both Greenland shark and Pacific sleeper shark characteristic haplotypes/alleles at various loci. Six sharks sampled in the Canadian Arctic, exhibited a Pacific sleeper shark cyt *b* haplotype but were homozygous for the Greenland shark alleles at both the ITS2 and RAG1 loci. An individual sampled in the temperate western Atlantic (i.e., “Nova Scotia” shark) exhibited a cyt *b* haplotype of a Pacific sleeper shark, homozygosity at ITS2 for the Pacific sleeper shark allele, but was heterozygous at RAG1, showing alleles for both Pacific sleeper and Greenland sharks at the RAG1 locus. Similarly, an individual sampled from the Gulf of Mexico showed a Pacific sleeper cyt *b* haplotype and homozygosity for the Pacific ITS2 allele, but also displayed heterozygosity for the Greenland shark and Pacific sleeper shark alleles at RAG1. Thus, although genetic sequences at the sampled loci tended to separate the somniosid species, evidence of genetic admixture between the two species was clear.

### 3.1 Demographic expansion

**Figure 3:**
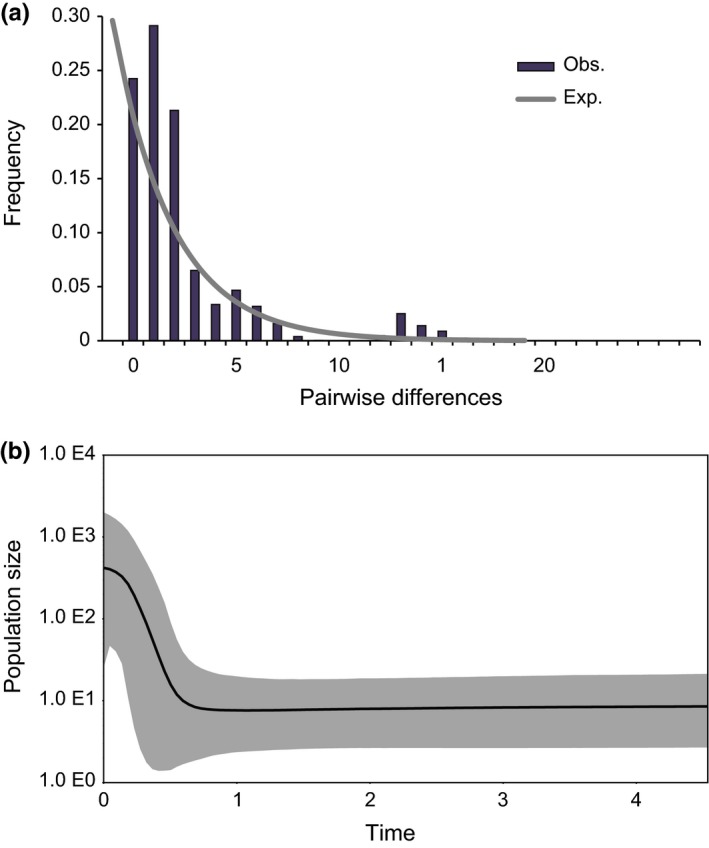
Genetic indicators of population expansion using the mtDNA cyt *b* data for *Somniosus microcephalus* individuals (including hybrids). (a) Mismatch distribution of pairwise nucleotide differences (dark gray columns) with expected curve fit to a model of population expansion (gray line); (b) Bayesian skyline plot for *S. microcephalus* using the HKY+I nucleotide substitution model

Tests for population demographic changes in Greenland sharks, performed with and without hybrids all corroborated the likely recent demographic expansion of the species. Genetic distance (*D*_a_) between Greenland sharks (including hybrids) and Pacific sleeper sharks was 0.01485. Tests for population demographic changes, with and without hybrid individuals included, showed significant negative Fu's *F* and Tajima's *D* values (*F*_hybrids_ = −35.903, *p* < .0001, *F*_no hybrids_ = −45.518, *p *<* *.01, *D*_hybrids_ = −1.95, *p *<* *.05, *D*_no hybrids_ = −2.19, *p *<* *.01, respectively), consistent with recent population expansion. Mismatch distribution analysis, outlining changes to species nucleotide diversity over time and effective population size (*N*_e_), also indicated a likely recent population expansion (Fig. 3a), while the BSP revealed a long period of population stability followed by a recent demographic expansion beginning around 0.5 Ma ago (Fig. 3b).

There was no significant genetic differentiation of *S. microcephalus* among geographic sampling sites with the exception of Cumberland Sound versus Maxwell Bay (*F*_ST_) and Maxwell Bay versus Nova Scotia (Φ_ST_, Table 2).

**Table 2:** Measures of genetic differentiation among *Somniosus* sampling sites. Φ_ST_ values (Tamura‐Nei; below diagonal), conventional *F*_ST_ based on haplotype frequency (above diagonal). All values non‐significant (*p* > .05), except in bold

Site	CS	DI	SV	GF	KP	IC	MB	NS	RB	SI
CS		0.0306	0.0111	−0.0800	−0.0664	−0.0152	**0.0380**	−0.0147	−0.0357	−0.0031
DI	0.0252		0.1661	−0.1250	0.0000	0.0698	0.2944	0.0924	−0.0193	0.0805
SV	−0.0090	0.1188		−0.0099	−0.0757	−0.0046	−0.0005	−0.0357	−0.0119	−0.0055
GF	−0.1161	−0.0461	−0.0784		−0.2000	−0.1009	0.0329	−0.0909	−0.1234	−0.0411
KP	−0.1297	−0.2026	−0.0299	−0.1999		−0.0855	0.0069	−0.1915	−0.1401	−0.0704
IC	−0.0072	0.1672	0.0085	−0.0966	−0.0713		0.0098	−0.0335	−0.0404	−0.0059
MB	−0.0089	0.1165	−0.0084	−0.1077	−0.0809	0.0139		0.0164	0.0400	0.0271
NS	0.1517	−0.0578	0.2396	−0.1484	−0.1786	0.2394	**0.1373**		−0.0667	−0.0319
RB	−0.0290	−0.0963	−0.0114	−0.1468	−0.2019	−0.0018	−0.0102	0.0438		−0.0303
SI	−0.0077	−0.0259	−0.0050	−0.1177	−0.1382	0.0028	−0.0030	0.0983	−0.0361	

### 3.2 Coalescent IM versus ILS analysis and divergence estimates

Across all isolation with migration (IM) models assessing coalescent‐based demographic parameter, strongest support was recovered from models with unequal migration rates between the species. The best models were characterized by coalescent migration rates equal to zero from Pacific sleeper sharks into Greenland sharks, while Greenland sharks into Pacific sleeper sharks migration rates were greater than zero. The best model also included varying long‐term effective population sizes for extant and ancestral populations, following AIC model selection tests. Following conversion of coalescent rates to “forward‐time,” results indicate asymmetrical migration whereby gene flow likely only occurs from Pacific sleeper sharks into Greenland sharks since the species diverged from one another.

### 3.3 mtDNA (cyt *b*) analysis

**Figure 4:**
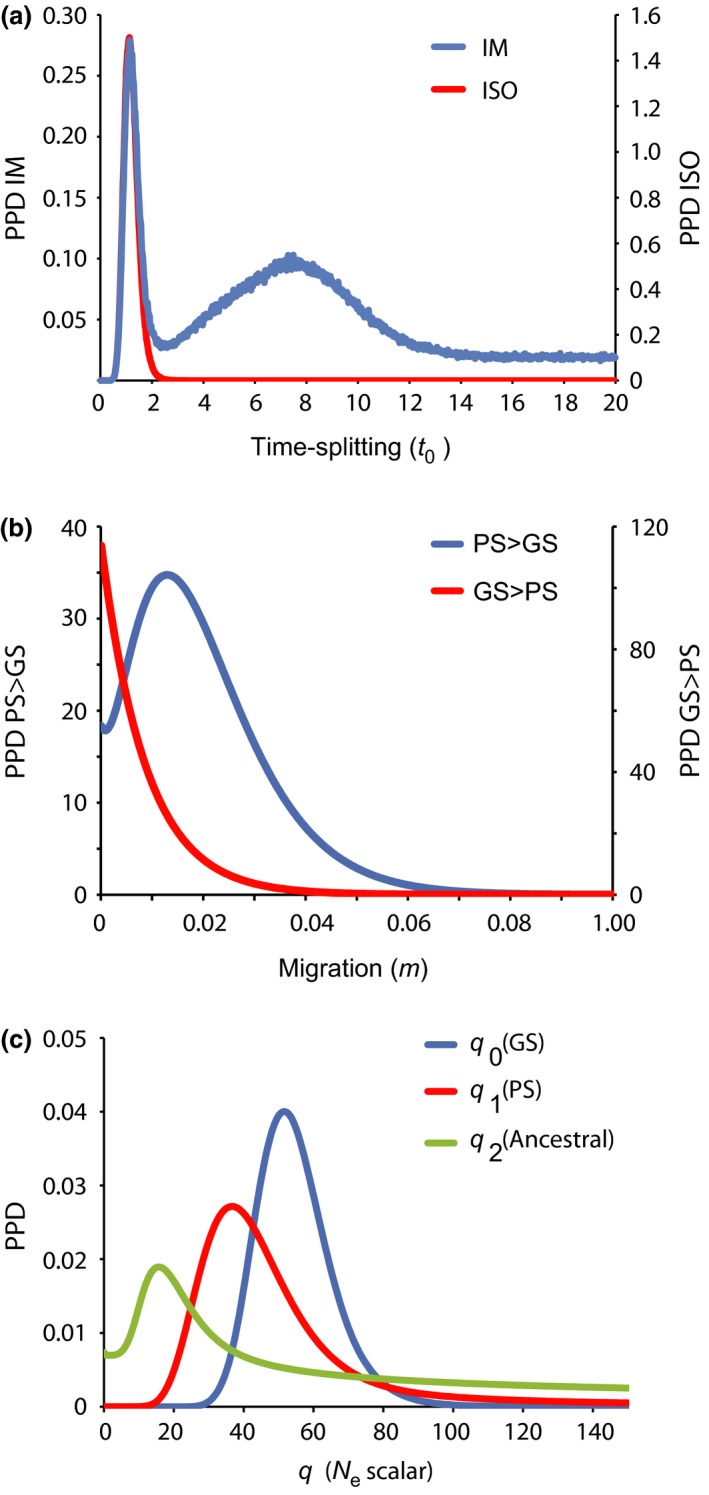
Marginal posterior probability density distributions of species‐split time (*t*_0_; panel a), migration rates (*m*; panel b) and long‐term effective population size (*N*_e_; panel c) as estimated in IMa2 using mtDNA (cyt *b*). Migration directions are presented forward in time. PPD, posterior probability density

Simulations of IM models (*m* prior upper bound = 0.2) and ISO (*m* = 0) models produced similar maximum‐likelihood estimates (hereafter MLEs) of species‐split time (*t*_0_) of 1.190 (Fig. 4a) and 1.130, respectively (see Table 3 for comparative split times in years). For the IM analyses, a second older and less probable peak was identified for *t*_0_ at 7.550 followed by a flat nonzero right‐end tail (Fig. 4a). Although overall migration rate parameters (forward‐time) were not significant following the Nielsen and Wakeley (2001) log‐likelihood ratio test, the population‐specific migration rate (2Nm; also forward‐time) for Pacific sleeper into Greenland shark was significantly different from zero (*p* < .05). Marginal MLE of migration were asymmetrical with *m*_PS>GS_ 0.01330 and *m*_GS>PS_ 0.00010 (Fig. 4b). Marginal unscaled estimates of *q* parameters and scaled *N*_e_ are presented in Table 3. Although generation times have not been reported for either *S. microcephalus* or *S. pacificus*, we applied a relatively short estimate based on a 20‐year age at first reproduction hypothesized for another squaliform species *Centroselachus crepidater* (Irvine, Stevens, & Laurenson, 2006; Veríssimo, McDowell, & Graves, 2011), and an approximated 150‐year generation length following the ~156 age at first reproduction hypothesized by Nielsen et al. (2016). The longer generation time scaled *N*_e_ estimates were approximately 13% smaller than the short‐generation time estimates (Table 3).

**Table 3:** (a) Time‐splitting estimates (*t*_0_) obtained from IMa2 for Isolation with Migration (IM) and Isolation only (ISO) models unscaled and scaled in millions years among *Somniosus microcephalus* and *Somniosus pacificus*, using published mtDNA substitution rates: mtDNA Conservative (Martin et al., 1992), mtDNA *Squalus* (Winchell et al., 2004), and mtDNA *Squatina* (Winchell et al., 2004). *Smi*,* Somniosus microcephalus*;* Spa*,* Somniosus pacificus*. (b) Comparative long‐term *N*_e_ estimates based on generation times of 20 years (*N*_e_20) and 150 years (*N*_e_150)

Unscaled		Conservative7.00 × 10^−10^		*Squalus* rate9.90 × 10^−10^		*Squatina* rate1.67 × 10^−9^	
IM	ISO	IM	ISO	IM	ISO	IM	ISO
(a) Time‐splitting *t*_0_							
1.15	1.13	2.34	2.30	1.65	1.63	0.98	0.96

Species	Unscaled	Conservative		*Squalus* rate		*Squatina* rate	
		*N*_e_20	*N*_e_150	*N*_e_20	*N*_e_150	*N*_e_20	*N*_e_150
(b) Long‐term *N*_e_							
*Smi*	51.30	1,304,945	173,993	922,662	173,993	548,077	73,077
*Spa*	36.75	934,829	124,644	660,971	88,129	392,628	52,350

### 3.4 Nuclear analysis

Nuclear divergence estimates between species were comparable to the cyt *b* coalescent analysis. An estimated split time of 3.16 Ma was found based on the single mutation difference between Greenland sharks and Pacific sleeper shark RAG1 alleles; while the ITS2 locus estimate was slightly younger at 2.52 Ma, based on the four mutation steps between both shark species.

## 4 DISCUSSION

Our genetic analyses clearly demonstrate the existence of two genetically differentiated mtDNA and nDNA lineages of *Somniosus* in the Northern hemisphere, largely consistent with the current recognized species ranges (MacNeil et al., 2012). However, cytonuclear discordance (initially reported among juvenile *S. microcephalus* in the Canadian Arctic, see Hussey et al., 2015) was present in eight individuals in this study; seven individuals from the known *S. microcephalus* distribution (MacNeil et al., 2012) and in an additional individual sampled well outside the known distribution (i.e., Gulf of Mexico). These data provide evidence for at least three classes of genetically admixed (i.e., hybrid) individuals that account for roughly 2% of the somniosid individuals in this study. Despite our dataset representing the largest sample size of Greenland sharks genetically surveyed to date, the elevated number of rare mtDNA haplotypes and the few individuals representing them, along with our failure to recover two previously reported Greenland shark haplotypes (Murray et al., 2008), suggests we have yet to capture the full extant genetic diversity of Greenland sharks. The hidden genetic diversity of Greenland shark notwithstanding the observed cyt *b* variation (i.e., high number of recovered haplotypes and the star‐like network pattern, mismatch distribution, and BSP) is consistent with a signal of demographic expansion following divergence from the ancestral somniosid. No obvious spatial genetic variation was observed (Table 2); however, the conspicuous Atlantic residency of all individuals carrying the pure‐type Greenland shark genetic signatures compared with the extant Pacific sleeper shark (and ancestral) lineages suggests speciation within, and likely adaptation to, the north Atlantic and/or Arctic environment.

The mixed genetic signatures among individuals identified in the field as Greenland shark could result from two possible processes: (1) ongoing interspecific hybridization (introgression upon secondary contact), or (2) the retention of ancestral DNA polymorphisms at the observed loci (incomplete lineage sorting—ILS). To attempt to distinguish between these two processes we applied IMa2 analyses on the mtDNA data. All IMa2 model‐fitting analysis indicated higher support for models including gene flow as the initial species split, and in all cases, this gene flow was asymmetrical with alleles originating in Pacific sleeper shark and migrating into Greenland shark. These results are consistent with a scenario of introgressive hybridization. This could explain the occurrence of Pacific sleeper shark mtDNA in Greenland sharks sampled in Scott Inlet in the Canadian Arctic (previously reported by Hussey et al., 2015) and found in other Arctic and sub‐Arctic areas such as Cumberland Sound and Maxwell Bay. The occurrence of admixed sharks in temperate and subtropical waters begs the question as to the source and origin of the Pacific sleeper shark alleles found in Greenland shark. While the Nova Scotia hybrid was caught within the distribution recognized for Greenland shark (MacNeil et al., 2012), the Gulf of Mexico shark was captured well outside this range, in the subtropical North Atlantic region. Moreover, the MAR‐Azores sample represents the first confirmed report of a complete Pacific sleeper shark genetic signature in the eastern Atlantic Ocean. These new findings suggest that DNA sequences currently reported as Pacific sleeper shark genetic signatures are not exclusive to the Pacific Ocean and that the common ancestor to both sharks likely exhibited a pan‐oceanic, deepwater distribution. Such a finding would also suggest that the divergence between the two species is, therefore, not likely linked to a single vicariant speciation event such as the rise of the Isthmus of Panama (see e.g., Bacon et al., 2015 and Marko, Eytan, & Knowlton, 2015 for a recent discussion). From the fossil record, the earliest appearance of sleeper sharks date to approximately 100 Ma ago with fossil somniosids found in Miocene deposits from Belgium and Italy (Kriwet & Klug, 2009). This suggests the Atlantic presence of somniosids prior to late Miocene cooling and subsequent onset of the Pleistocene glacial periods.

Estimates of species‐split (divergence) time for Greenland shark and Pacific sleeper shark using mtDNA did not show a consistent time line for IM and ILS models across a range of substitution rates. The observed bimodal *t*_0_ curve, however, is consistent with a “two attractors” hypothesis described as “a recent signal of no migration, and an older signal with higher migration” (J. Hey as cited in Choleva et al., 2014). Application of a broadly based conservative substitution rate (Martin et al., 1992) and more probable substitution rates (see Table 3) identified that an IM‐based estimated species‐split time using the cyt *b* locus occurred during the Quaternary period, roughly 0.98 Ma ago to 2.34 Ma ago. This time frame is consistent with the onset of the Pleistocene epoch, which may have played an initiating role in the speciation trajectories of extant sleeper sharks in the Northern hemisphere. Analysis of nuclear data alone, though exhibiting only low diversity and differentiation, also supports a more recent split. Analyses not accounting for interspecific hybridization (e.g., ILS runs, and BSP) further support more recent processes (i.e., Pleistocene) as exerting demographic impacts including increased *N*_e_ as suggested by the BSP (Fig. 3b) around 0.5 Ma.

### 4.1 Ice‐olation with migration hypothesis

We introduce the hypothesis of oscillatory vicariance with secondary contact—herein referred to as Ice‐olation with Migration. If we consider that the ancestral sleeper shark was a deepwater species that was constrained to this habitat via temperature regimes, this species likely occupied a pan‐oceanic distribution prior to the late Miocene, but only in the deep ocean. This is also consistent with the occurrence of somniosids in tropical and temperate regions where they are typically observed at depths greater than 1,000 m and reportedly down to 2,200 m (Benz et al., 2007; Campana, Fisk, & Klimley, 2015; Herdendorf & Berra, 1995), and in contrast to somniosids sampled in the Arctic and northern Quebec, where satellite telemetry data have shown sharks occurring from the surface down to approximately 1,500 m (Campana et al., 2015; Fisk, Lydersen, & Kovacs, 2012; Stokesbury, Harvey‐Clark, Gallant, Block, & Myers, 2005).

From the late Miocene, and into the Quaternary, a drastic reduction in global temperatures occurred. This decline in sea temperature resulted in sea ice formation in polar regions, including the formation of ice shelves up to 1 km in thickness (Fastook & Hughes, 2013; Grosswald & Hughes, 1999; Polyak, Edwards, Coakley, & Jakobsson, 2001). This ice thickness coupled with the submerged mountainous terrain of the Arctic, may have effectively constrained population connectivity of ancestral somniosids between the Arctic sub‐basins/marginal seas and the remaining pan‐oceanic distribution. In support of the Arctic sub‐basin isolation, glacial scouring patterns along the Lomonosov Ridge are consistent with ice shelf formations (Polyak et al., 2001; Stein et al., 2016), and it is likely that similar constraints to connectivity occurred among the Arctic and Atlantic Ocean at the East Greenland Rift Basin (Vigdorchik, 1979; Vigdorchik & Vinkovetsky, 1976). The alternating advance and retreat glaciation stages, and repeated ice shelf formation and grounding on Arctic shelves would have yielded transient periods of isolation—typically 50–100 k years in duration—long enough to initiate genetic divergence, but likely not long enough to facilitate complete reproductive isolation. Subsequent interglacial periods restored connectivity among basins. Similar processes have been proposed regarding the 100 k year scale of isolation of marine fishes across the Sunda Shelf followed by subsequent connectivity (Reece, Bowen, Smith, & Larson, 2010).

During cooling periods, polar regions may have also exhibited a more uniform thermal profile than the stratified profiles of the temperate and tropical regions. Combined, these cooler temperatures and the uniformity of vertical temperature profiles in the Arctic may have facilitated an expansion in available habitat for sleeper sharks, from deep waters to shallow coastal habitats. Habitat expansion would have concurrently provided access to a diverse array of prey, including marine mammals. The demographic expansion suggested by the mismatch analysis and the BSP, and the more than doubling of long‐term *N*_e_ estimates (from IMa2 analyses) from ancestral sharks to the current species is consistent with the expansion of available habitat and prey resources for somniosid species in the Arctic region.

### 4.2 Population expansion and adaptation in the Arctic

Ecological specialization has been proposed as a driver for marine speciation (Corrigan & Beheregaray, 2009; Kashiwagi et al., 2012; Morin et al., 2010; Wang, Chou, & White,^1999^) and is a plausible explanation for the demographic expansion of sleeper sharks in the Arctic region. Greenland sharks are considered generalists and opportunistic predators feeding on a broad range of benthic and pelagic teleost and cephalopod prey (MacNeil et al., 2012). Marine mammals including ringed‐seal (*Pusa hispida*) have been recovered from the stomach contents of Arctic Greenland sharks (Fisk, Tittlemier, Pranschke, & Norstrom, 2002; Leclerc et al., 2012; Lydersen, Fisk, & Kovacs, 2016; McMeans, Svavarsson, Dennard, & Fisk, 2010; Nielsen, Hedeholm, Simon, & Steffensen, 2014; Ridoux, Hall, Steingrimsson, & Olafsson, 1998), as well as in the stomachs of Pacific sleeper sharks at high latitudes (Bright, 1959; Horning & Mellish, 2014; Hulbert, Sigler, & Lunsford, 2001), identifying both species as high trophic level feeders (Hussey et al., 2014). When considering the global distribution of shark and pinniped diversity, an inverse relationship occurs, with the highest shark diversity at tropical latitudes where pinniped diversity is low, and few large shark species, including the Greenland and Pacific sleeper shark, occurring at high latitudes where pinniped diversity is highest (Ferguson, Higdon, Tallman, Fisk, & Hussey, 2014). The diversity and abundance of marine mammals in the Arctic might have served as novel food sources for sleeper sharks during their colonization of the Arctic environment.

## 5 CONCLUSION

Our results show that the nearly morphologically identical sister species Greenland shark and Pacific sleeper shark can be genetically distinguished using a combination of mtDNA and nuclear markers, but interspecific hybridization (introgression) between these two species is probable and has likely been ongoing since initial divergence. Our results confirm the fourth reported instance of interspecific hybridization among elasmobranchs (Hussey et al., 2015; see also Morgan et al., 2012; Walter et al., 2014; Cruz et al., 2015), which is likely more common among this group of fishes than previously thought. Our findings, considering a range of substitution rates for the genetic markers employed here, are consistent with a more recent Greenland shark and Pacific sleeper shark speciation event occurring between 1 and 2.34 Ma, more recently than the closure of the Isthmus of Panama. Our estimates of long‐term *N*_e_s also indicate slightly higher sizes for Greenland sharks compared to Pacific sleeper sharks. However, recent radiocarbon analysis of *S. microcephalus* eye lenses has concluded that Greenland sharks may not reach maturity until 150 years of age, and possibly live as long as 400 years (Nielsen et al., 2016). If accurate, this would mean that effective population sizes are but a fraction of our 20‐year estimates, and using the *N*_e_ estimates from the 150‐year generation time of Nielsen et al. (2016) is likely more appropriate (Table 3). The lack of geographic structuring for Greenland sharks across the Canadian Arctic and beyond, but with the recurring hybridization with Pacific sleeper sharks at the edge of its distribution in warmer waters, suggests that a continuing warming Arctic may further reduce the genetic integrity of both species. Future research focused on broad‐scale population‐level connectivity across the range of *S. microcephalus* is required to clarify the consequences of past climatic and present‐day environmental changes on the hybridization rates and evolutionary trajectory of these species.

## CONFLICT OF INTEREST

None declared.

## References

[ece33325-bib-0001] Andersen, L. W. , & Born, E. W. (2000). Identifications of two genetically different sub‐populations of Atlantic walruses (*Odobenus rosmarus rosmarus*) in West and Northwest Greenland. Canadian Journal of Zoology, 78, 1999–2009.

[ece33325-bib-0002] Andersen, L. W. , Born, E. W. , Gjertz, I. , Wiig, Ø. , Holm, L. , & Bendixen, C. (1998). Population structure and gene flow of the Atlantic walrus (*Odobenus rosmarus rosmarus*) in the eastern Atlantic Arctic based on mitochondrial DNA and microsatellite variation. Molecular Ecology, 7, 1323–1336. 9787444 10.1046/j.1365-294x.1998.00455.x

[ece33325-bib-0003] Avise, J. C. (1992). Molecular population structure and biogeographic history of a regional fauna: A case history with lessons for conservative biology. Oikos, 63, 62–76.

[ece33325-bib-0004] Bacon, C. D. , Silvestro, D. , Jaramillo, C. , Smith, B. T. , Chakrabarty, P. , & Antonelli, A. (2015). Biological evidence supports an early and complex emergence of the Isthmus of Panama. Proceedings of the National Academy of Sciences of the United States of America, 112, 6110–6115. 25918375 10.1073/pnas.1423853112 PMC4434730

[ece33325-bib-0005] Benz, G. W. , Hoffmayer, E. R. , Driggers, E. R. , Allen, W. B. , Bishop, D. , Lougan, E. , & Brown, D. A. (2007). First record of sleeper shark in the western Gulf of Mexico and comments on taxonomic uncertainty within *Somniosus* (*Somniosus*). Bulletin of Marine Science, 80, 343–351.

[ece33325-bib-0006] Bremer, J. R. A. , Mejuto, J. , Greig, T. W. , & Ely, B. (1996). Global population structure of the swordfish (*Xiphias gladius* L) as revealed by analysis of the mitochondrial DNA control region. Journal of Experimental Marine Biology and Ecology, 197, 295–310.

[ece33325-bib-0007] Briggs, J. C. (2006). Proximate sources of marine biodiversity. Journal of Biogeography, 33, 1–10.

[ece33325-bib-0008] Bright, D. B. (1959). The occurrence and food of the sleeper shark, *Somniosus pacificus*, in a central Alaska bay. Copeia, 1, 76–77.

[ece33325-bib-0009] Campana, S. E. , Fisk, A. T. , & Klimley, A. P. (2015). Movements of Arctic and northwest Atlantic Greenland sharks (*Somniosus microcephalus*) monitored with archival satellite pop‐up tags suggest long‐range migrations. Deep Sea Research II, 115, 109–115.

[ece33325-bib-0500] Chernova, N. V. , Smirnovab, E. V. , Raskhozhevab, E. V. (2015). First record of the Greenland shark Somniosus microcephalus (Squaliformes: Somniosidae) in the Siberian Arctic with notes on its distribution and biology. Journal of Ichthyology, 55, 827–835.

[ece33325-bib-0010] Choleva, L. , Musilova, Z. , Kohoutova‐Sediva, A. , Paces, J. , Rab, P. , & Janko, K. (2014). Distinguishing between incomplete lineage sorting and genomic introgressions: Complete fixation of allospecific mitochondrial DNA in a sexually reproducing fish (*Cobitis*; Teleostei), despite clonal reproduction of hybrids. PLoS ONE, 9, e80641. 24971792 10.1371/journal.pone.0080641 PMC4074047

[ece33325-bib-0011] Clarke, L. M. , Munch, S. B. , Thorrold, S. R. , & Conover, D. O. (2010). High connectivity among locally adapted populations of a marine fish (*Menidia menidia*). Ecology, 91, 3526–3537. 21302825 10.1890/09-0548.1

[ece33325-bib-0012] Clement, M. , Posada, D. , & Crandall, K. A. (2000). TCS: A computer program to estimate gene genealogies. Molecular Ecology, 9, 1657–1659. 11050560 10.1046/j.1365-294x.2000.01020.x

[ece33325-bib-0013] Corrigan, S. , & Beheregaray, L. B. (2009). A recent shark radiation: Molecular phylogeny, biogeography and speciation of wobbegong sharks (Orectolobidae). Molecular Phylogenetics and Evolution, 52, 205–216. 19303452 10.1016/j.ympev.2009.03.007

[ece33325-bib-0014] Cowman, P. F. , & Bellwood, D. R. (2013). Vicariance across major marine biogeographic barriers: Temporal concordance and the relative intensity of hard versus soft barriers. Proceedings of the Royal Society of London B: Biological Sciences, 280, 20131541. 10.1098/rspb.2013.1541 PMC3757981 23945690

[ece33325-bib-0015] Coyne, J. A. , & Orr, H. A. (2004). Speciation. Sunderland, MA: Sinauer Associates.

[ece33325-bib-0016] Crow, K. D. , Munehara, H. , & Bernardi, G. (2010). Sympatric speciation in a genus of marine reef fishes. Molecular Ecology, 19, 2089–2105. 20345669 10.1111/j.1365-294X.2010.04611.x

[ece33325-bib-0017] Cruz, V. P. , Vera, M. , Mendonça, F. F. , Pardo, B. G. , Martinez, P. , Oliveira, C. , & Foresti, F. (2015). First identification of interspecies hybridization in the freshwater stingrays *Potamotrygon motoro* and *P. falkneri* (Myliobatiformes, Potamotrygonidae). Conservation Genetics, 16, 241–245.

[ece33325-bib-0018] Darriba, D. , Taboada, G. L. , Doallo, R. , & Posada, D. (2012). jModelTest 2: More models, new heuristics and parallel computing. Nature Methods, 9, 772. 10.1038/nmeth.2109 PMC4594756 22847109

[ece33325-bib-0019] da Silva Ferrette, B. L. , Mendonça, F. F. , Coelho, R. , de Oliveira, P. G. V. , Hazin, F. H. V. , Romanov, E. V. , et al. (2015). High connectivity of the crocodile shark between the Atlantic and southwest Indian Oceans: Highlights for conservation. PLoS ONE, 10, e0117549. 25689742 10.1371/journal.pone.0117549 PMC4331560

[ece33325-bib-0020] Drummond, A. J. , Rambaut, A. , Shapiro, B. , & Pybus, O. G. (2005). Bayesian coalescent inference of past population dynamics from molecular sequences. Molecular Biology and Evolution, 22, 1185–1192. 15703244 10.1093/molbev/msi103

[ece33325-bib-0021] Drummond, A. J. , Suchard, M. A. , Xie, D. , & Rambaut, A. (2012). Bayesian phylogenetics with BEAUti and the BEAST 1.7. Molecular Biology and Evolution, 29, 1969–1973. 22367748 10.1093/molbev/mss075 PMC3408070

[ece33325-bib-0022] Eizaguirre, C. , & Baltazar‐Soares, M. (2009). Evolutionary conservation‐evaluating the adaptive potential of species. Evolutionary Applications, 7, 963–967.

[ece33325-bib-0023] Excoffier, L. , Laval, G. , & Schneider, S. (2005). Arlequin ver. 3.0: An integrated software package for population genetics data analysis. Evolutionary Bioinformatics Online, 1, 47–50. PMC2658868 19325852

[ece33325-bib-0024] Eytan, R. I. , & Hellberg, M. E. (2010). Nuclear and mitochondrial sequence data reveal and conceal different demographic histories and population genetic processes in Caribbean reef fishes. Evolution, 64, 3380–3397. 20584072 10.1111/j.1558-5646.2010.01071.x

[ece33325-bib-0025] Fastook, J. L. , & Hughes, T. J. (2013). New perspectives on paleoglaciology. Quaternary Science Reviews, 80, 169–194.

[ece33325-bib-0026] Ferguson, S. , Higdon, J. , Tallman, R. , Fisk, A. T. , & Hussey, N. E. (2014). The ghost of competition past: Body size, trophic ecology, diversity and distribution of global shark and pinniped species. Journal of Marine Animals and their Ecology, 7, 23–39.

[ece33325-bib-0027] Fisk, A. T. , Lydersen, C. , & Kovacs, K. M. (2012). Archival pop‐off tracking of Greenland sharks *Somniosus microcephalus* in the High Arctic waters of Svalbard, Norway. Marine Ecology Progress Series, 468, 255–265.

[ece33325-bib-0028] Fisk, A. T. , Tittlemier, S. A. , Pranschke, J. L. , & Norstrom, R. J. (2002). Using anthropogenic contaminants and stable isotopes to assess the feeding ecology of Greenland sharks. Ecology, 83, 2162–2172.

[ece33325-bib-0029] Gaither, M. R. , Bowen, B. W. , Toonen, R. J. , Planes, S. , Messmer, V. , Earle, J. , & Robertson, D. R. (2010). Genetic consequences of introducing allopatric lineages of bluestriped snapper (*Lutjanus kasmira*) to Hawaii. Molecular Ecology, 19, 1107–1121. 20163550 10.1111/j.1365-294X.2010.04535.x

[ece33325-bib-0030] Griffiths, A. M. , Sims, D. W. , Cotterell, S. P. , El Nagar, A. , Ellis, J. R. , Lynghammar, A. , … Genner, M. J. (2010). Molecular markers reveal spatially segregated cryptic species in a critically endangered fish, the common skate (*Dipturus batis*). Proceedings of the Royal Society of London B: Biological Sciences, 277, 1497–1503. 10.1098/rspb.2009.2111 PMC2871835 20106849

[ece33325-bib-0031] Grosswald, M. G. , & Hughes, T. (1999). The case for an ice shelf in the Pleistocene Arctic Ocean. Polar Geography and Geology, 23, 23–54.

[ece33325-bib-0032] Hellberg, M. E. (2009). Gene flow andiIsolation among populations of marine animals. Annual Review of Ecology, Evolution, and Systematics, 40, 291–310.

[ece33325-bib-0033] Herdendorf, C. E. , & Berra, T. M. (1995). A Greenland shark from the wreck of the SS Central America at 2,200 meters. Transactions of the American Fisheries Society, 124, 950–953.

[ece33325-bib-0034] Hey, J. (2007). IMa documentation. Retrieved from http://genfaculty.rutgers.edu/hey/software.

[ece33325-bib-0035] Hey, J. (2010a). Isolation with migration models for more than two populations. Molecular Biology and Evolution, 27, 905–920. 19955477 10.1093/molbev/msp296 PMC2877539

[ece33325-bib-0036] Hey, J. (2010b). The divergence of chimpanzee species and subspecies as revealed in multipopulation isolation‐with‐migration analyses. Molecular Biology and Evolution, 27, 921–933. 19955478 10.1093/molbev/msp298 PMC2877540

[ece33325-bib-0037] Hey, J. , & Nielsen, R. (2007). Integration within the Felsenstein equation for improved Markov chain Monte Carlo methods in population genetics. Proceeding of the National Academy of Sciences of the United States of America, 104, 2785–2790. 10.1073/pnas.0611164104 PMC1815259 17301231

[ece33325-bib-0038] Hickerson, M. J. , & Meyer, C. (2008). Testing comparative phylogeographic models of marine vicariance and dispersal using a hierarchical Bayesian approach. BMC Evolutionary Biology, 8, 322. 19038027 10.1186/1471-2148-8-322 PMC2614435

[ece33325-bib-0039] Hinojosa‐Alvarez, S. , Walter, R. P. , Diaz‐Jaimes, P. , Galván‐Magaña, F. , & Paig‐Tran, E. M. (2016). A potential third Manta Ray species near the Yucatán Peninsula? Evidence for a recently diverged and novel genetic Manta group from the Gulf of Mexico. PeerJ, 4, e2586. 27833795 10.7717/peerj.2586 PMC5101608

[ece33325-bib-0040] Horne, J. B. , van Herwerden, L. , Choat, H. J. , & Robertson, D. R. (2008). High population connectivity across the Indo‐Pacific: Congruent lack of phylogeographic structure in three reef fish congeners. Molecular Phylogenetics and Evolution, 49, 629–638. 18804542 10.1016/j.ympev.2008.08.023

[ece33325-bib-0041] Horning, M. , & Mellish, J. E. (2014). In cold blood: Evidence of Pacific sleeper shark (*Somniosus pacificus*) predation on Steller sea lions (*Eumetopias jubatus*) in the Gulf of Alaska. Fisheries Bulletin, 112, 297–310.

[ece33325-bib-0042] Hulbert, L. , Sigler, M. F. , & Lunsford, C. R. (2001). Pacific sleeper shark predation on Steller sea lions In DeMaster D. C., & Atkinson S. C. (Eds.), Is it food II (pp. 67–69). Seward, AL: University of Alaska Sea Grant College Program.

[ece33325-bib-0043] Hussey, N. E. , Cosandey‐Godin, A. , Walter, R. P. , Hedges, K. J. , VanGerwen‐Toyne, M. , Barkley, A. N. , et al. (2015). Juvenile Greenland sharks *Somniosus microcephalus* (Bloch and Schneider, 1801) in the Canadian Arctic. Polar Biology, 38, 493–504.

[ece33325-bib-0044] Hussey, N. E. , MacNeil, M. A. , McMeans, B. C. , Olin, J. A. , Dudley, S. F. J. , Cliff, G. , … Fisk, A. T. (2014). Rescaling the trophic framework of marine food webs. Ecology Letters, 17, 239–250. 24308860 10.1111/ele.12226 PMC3912912

[ece33325-bib-0046] Ingram, T. (2011). Speciation along a depth gradient in a marine adaptive radiation. Proceedings of the Royal Society of London B, 278, 613–618. 10.1098/rspb.2010.1127 PMC3025674 20810434

[ece33325-bib-0047] Irvine, S. B. , Stevens, J. D. , & Laurenson, J. B. (2006). Surface bands on deepwater squalid dorsal‐fin spines: An alternative method for aging *Centroselachus crepidater* . Canadian Journal of Fisheries and Aquatic Sciences, 63, 617–627.

[ece33325-bib-0048] Kashiwagi, T. , Marshall, A. D. , Bennett, M. B. , & Ovenden, J. R. (2012). The genetic signature of recent speciation in manta rays (*Manta alfredi* and *M. birostris*). Molecular Phylogenetics and Evolution, 64, 212–218. 22503670 10.1016/j.ympev.2012.03.020

[ece33325-bib-0049] Kinlan, B. P. , & Gaines, S. D. (2003). Propagule dispersal in marine and terrestrial environments: A community perspective. Ecology, 84, 2007–2020.

[ece33325-bib-0050] Knowlton, N. , & Weigt, L. A. (1998). New dates and new rates for divergence across the Isthmus of Panama. Proceedings of the Royal Society of London B, 265, 2257–2263.

[ece33325-bib-0051] Kriwet, J. , & Klug, S. (2009). Fossil record and origin of squaliform sharks (Chondrichthyes, Neoselachii) In Gallucci V., McFarlane G., & Bargmann G. (Eds.), Biology and management of dogfish sharks (pp. 19–38). Bethesda, MD: American Fisheries Society.

[ece33325-bib-0052] Leclerc, L. M. E. , Lydersen, C. , Haug, T. , Bachmann, L. , Fisk, A. T. , & Kovacs, K. M. (2012). A missing piece in the Arctic food web puzzle? Stomach contents of Greenland sharks sampled in Svalbard, Norway. Polar Biology, 35, 1197–1208.

[ece33325-bib-0053] Librado, P. , & Rozas, J. (2009). DnaSP v5: A software for comprehensive analysis of DNA polymorphism data. Bioinformatics, 25, 1451–1452. 19346325 10.1093/bioinformatics/btp187

[ece33325-bib-0054] Lydersen, C. , Fisk, A. T. , & Kovacs, K. M. (2016). A review of Greenland shark (*Somniosus microcephalus)* studies in the Kongsfjorden area, Svalbard Norway. Polar Biology, 39, 2169–2178.

[ece33325-bib-0055] Mach, M. E. , Sbrocco, E. J. , Hice, L. A. , Duffy, T. A. , Conover, D. O. , & Barber, P. H. (2011). Regional differentiation and post‐glacial expansion of the Atlantic silverside, *Menidia menidia,* an annual fish with high dispersal potential. Marine Biology, 158, 515–530. 24391257 10.1007/s00227-010-1577-3 PMC3873031

[ece33325-bib-0056] MacNeil, M. A. , McMeans, B. C. , Hussey, N. E. , Vecsei, P. , Svavarsson, J. , Kovacs, K. M. , et al. (2012). Biology of the Greenland shark *Somniosus microcephalus* Bloch and Schneider, 1801. Journal of Fish Biology, 80, 991–1018. 22497371 10.1111/j.1095-8649.2012.03257.x

[ece33325-bib-0057] Marko, P. B. , Eytan, R. I. , & Knowlton, N. (2015). Do large molecular sequence divergences imply an early closure of the Isthmus of Panama? Proceedings of the National Academy of Sciences of the United States of America, 112, E5766. 26489657 10.1073/pnas.1515048112 PMC4629346

[ece33325-bib-0059] Martin, A. P. , Naylor, G. J. P. , & Palumbi, S. R. (1992). Rates of mitochondrial DNA evolution in sharks are slow compared with mammals. Nature, 357, 153–155. 1579163 10.1038/357153a0

[ece33325-bib-0060] Martin, A. P. , & Palumbi, S. R. (1993). Body size, metabolic‐rate, generation time, and the molecular clock. Proceedings of the National Academy of Sciences of the United States of America, 90, 4087–4091. 8483925 10.1073/pnas.90.9.4087 PMC46451

[ece33325-bib-0061] McMeans, B. C. , Svavarsson, J. , Dennard, S. , & Fisk, A. T. (2010). Diet and resource use among Greenland sharks (*Somniosus microcephalus*) and teleosts sampled in Icelandic waters, using d13C, d15N, and mercury. Canadian Journal of Fisheries and Aquatic Sciences, 67, 1428–1438.

[ece33325-bib-0062] Miglietta, M. P. , Faucci, A. , & Santini, F. (2011). Speciation in the sea: Overview of the symposium and discussion of future directions. Integrative and Comparative Biology, 51, 449–455. 21593140 10.1093/icb/icr024

[ece33325-bib-0063] Montoya‐Burgos, J. I. (2003). Historical biogeography of the catfish genus *Hypostomus* (Siluriformes: Loricariidae), with implications on the diversification of Neotropical ichthyofauna. Molecular Ecology, 12, 1855–1867. 12803637 10.1046/j.1365-294x.2003.01857.x

[ece33325-bib-0064] Moore, J. S. , Gow, J. L. , Taylor, E. B. , & Hendry, A. P. (2007). Quantifying the constraining influence of gene flow on adaptive divergence in the lake‐stream threespine stickleback system. Evolution, 61, 2015–2026. 17683442 10.1111/j.1558-5646.2007.00168.x

[ece33325-bib-0065] Morgan, J. A. T. , Harry, A. , Welch, D. , Street, R. , White, J. , Geraghty, P. T. , et al. (2012). Detection of interspecies hybridisation in Chondrichthyes: Hybrids (hybrid offspring) between Australian (*Carcharhinus tilstoni*) and common blacktip shark (*C. limbatus*) found in an Australian fishery. Conservation Genetics, 13, 455–463.

[ece33325-bib-0066] Morin, P. A. , Archer, F. I. , Foote, A. D. , Vilstrup, J. , Allen, E. E. , Wade, P. , et al. (2010). Complete mitochondrial genome phylogeographic analysis of killer whales (*Orcinus orca*) indicates multiple species. Genome Research, 20, 908–916. 20413674 10.1101/gr.102954.109 PMC2892092

[ece33325-bib-0067] Murray, B. M. , Wang, J. Y. , Yang, S. C. , Stevens, J. D. , Fisk, A. , & Svararsson, J. (2008). Mitochondrial cytochrome b variation in sleeper sharks (Squaliformes: Somniosidae). Marine Biology, 153, 1015–1022.

[ece33325-bib-0068] Nielsen, J. , Hedeholm, R. B. , Heinemeier, J. , Bushnell, P. G. , Christiansen, J. S. , Olsen, J. , et al. (2016). Eye lens radiocarbon reveals centuries of longevity in the Greenland shark (*Somniosus microcephalus*). Science, 353, 702–704. 27516602 10.1126/science.aaf1703

[ece33325-bib-0069] Nielsen, J. , Hedeholm, R. B. , Simon, M. , & Steffensen, J. F. (2014). Distribution and feeding ecology of the Greenland shark (*Somniosus microcephalus*) in Greenland waters. Polar Biology, 37, 37–46.

[ece33325-bib-0070] Nielsen, R. , & Wakeley, J. (2001). Distinguishing migration from isolation: A Markov chain Monte Carlo approach. Genetics, 158, 885–896. 11404349 10.1093/genetics/158.2.885 PMC1461674

[ece33325-bib-0071] Norris, R. D. , & Hull, P. M. (2012). The temporal dimension of marine speciation. Evolutionary Ecology, 26, 393–415.

[ece33325-bib-0072] Nosil, P. (2008). Speciation with gene flow may be common. Molecular Ecology, 17, 2103–2106. 18410295 10.1111/j.1365-294X.2008.03715.x

[ece33325-bib-0073] O'Corry‐Crowe, G. (2008). Climate change and the molecular ecology of Arctic marine mammals. Ecological Applications, 18, S56–S76. 18494363 10.1890/06-0795.1

[ece33325-bib-0074] O'Corry‐Crowe, G. , Lydersen, C. , Heide‐Jørgensen, M. P. , Hansen, L. , Mukhametov, L. M. , Dove, O. , & Kovacs, K. M. (2010). Population genetic structure and evolutionary history of North Atlantic beluga whales (*Delphinapterus leucas*) from west Greenland, Svalbard and the White Sea. Polar Biology, 33, 1179–1194.

[ece33325-bib-0075] O'Corry‐Crowe, G. , Mahoney, A. R. , Suydam, R. , Quakenbush, L. , Whiting, A. , Lowry, L. , & Harwood, L. (2016). Genetic profiling links changing sea‐ice to shifting beluga whale migration patterns. Biology Letters, 12, 20160404 https://doi.org/10.1098/rsbl.2016.0404

[ece33325-bib-0076] O'Corry‐Crowe, G. M. , Suydam, R. S. , Rosenberg, A. , Frost, K. J. , & Dizon, A. E. (1997). Phylogeography, population structure and dispersal patterns of the beluga whale *Delphinapterus leucas* in the western Nearctic revealed by mitochondrial DNA. Molecular Ecology, 6, 955–970.

[ece33325-bib-0077] Palo, J. U. (2003). Genetic diversity and phylogeography of landlocked seals. Dissertation, University of Helsinki, Helsinki, Finland.

[ece33325-bib-0078] Palsbøll, P. J. , Heide‐Jorgensen, M. P. , & Dietz, R. (1997). Population structure and seasonal movements of narwhals, *Monodon monoceros*, determined from mtDNA analysis. Heredity, 78, 284–292. 9119704 10.1038/hdy.1997.43

[ece33325-bib-0079] Palumbi, S. R. (1994). Genetic divergence, reproductive isolation, and marine speciation. Annual Review of Ecology and Systematics, 25, 547–572.

[ece33325-bib-0080] Palumbi, S. R. (2004). Marine reserves and ocean neighborhoods: The spatial scale of marine populations and their management. Annual Review of Environment and Resources, 29, 31–68.

[ece33325-bib-0082] Pelletier, F. , Garant, D. , & Hendry, A. P. (2009). Eco‐evolutionary dynamics. Proceedings of the Royal Society of London B: Biological Sciences, 364, 1483–1489. 10.1098/rstb.2009.0027 PMC2690510 19414463

[ece33325-bib-0084] Polyak, L. , Edwards, M. H. , Coakley, B. J. , & Jakobsson, M. (2001). Ice shelves in the Pleistocene Arctic Ocean inferred from glaciogenic deep‐sea bedforms. Nature, 410, 453–457. 11260709 10.1038/35068536

[ece33325-bib-0085] Puebla, O. (2009). Ecological speciation in marine *v*. freshwater fishes. Journal of Fish Biology, 75, 960–996. 20738594 10.1111/j.1095-8649.2009.02358.x

[ece33325-bib-0086] Rambaut, A. , & Drummond, A. J. (2007). Tracer v. 1.5. Retrieved from http://tree.bio.ed.ac.uk/software/tracer.

[ece33325-bib-0087] Rapoport, E. H. (1994). Remarks on marine and continental biogeography—An areographical viewpoint. Philosophical Transactions of the Royal Society of London Series B Biological Science, 343, 71–78.

[ece33325-bib-0088] Reece, J. S. , Bowen, B. W. , Smith, D. G. , & Larson, A. F. (2010). Molecular phylogenetics of moray eels (Muraenidae) dem‐onstrates multiple origins of shell‐crushing jaw (*Gymnomuraena*,* Echidna*) and multiple colonizations of the Atlantic Ocean. Molecular Phylogenetics and Evolution, 57, 829–835. 20674752 10.1016/j.ympev.2010.07.013

[ece33325-bib-0089] Ridoux, V. , Hall, A. J. , Steingrimsson, G. , & Olafsson, G. (1998). An inadvertent homing experiment with a young ringed seal*, Phoca hispida* . Marine Mammal Science, 14, 883–888.

[ece33325-bib-0090] Rocha, L. A. , Robertson, D. R. , Roman, J. , & Bowen, B. W. (2005). Ecological speciation in tropical reef fishes. Proceedings of the Royal Society of London B: Biological Sciences, 272, 573–579. 10.1098/2004.3005 PMC1564072 15817431

[ece33325-bib-0091] Roy, D. , Hardie, D. C. , Treble, M. A. , Reist, J. D. , & Ruzzante, D. E. (2014). Evidence supporting panmixia in Greenland halibut (*Reinhardtius hippoglossoides*) in the Northwest Atlantic. Canadian Journal of Fisheries and Aquatic Sciences, 71, 763–774.

[ece33325-bib-0092] Roy, D. , Hurlbut, T. R. , & Ruzzante, D. E. (2012). Biocomplexity in a demersal exploited fish, white hake (*Urophycis tenuis*): Depth‐related structure and inadequacy of current management approaches. Canadian Journal of Fisheries and Aquatic Sciences, 69, 415–429.

[ece33325-bib-0093] Schmidt, J. V. , Schmidt, C. L. , Ozer, F. , Ernst, R. E. , Feldheim, K. A. , Ashley, M. V. , & Levine, M. (2009). Low genetic differentiation across three major ocean populations of the whale shark, *Rhincodon typus* . PLoS ONE, 4, e4988. 19352489 10.1371/journal.pone.0004988 PMC2662413

[ece33325-bib-0094] Seehausen, O. , Terai, Y. , Magalhaes, I. S. , Carleton, K. L. , Mrosso, H. D. J. , Miyagi, R. , … Okada, N. (2008). Speciation through sensory drive in cichlid fish. Nature, 455, 620–626. 18833272 10.1038/nature07285

[ece33325-bib-0095] Stein, R. , Fahl, K. , Schreck, M. , Knorr, G. , Niessen, F. , Forwick, M. , et al. (2016). Evidence for ice‐free summers in the late Miocene central Arctic Ocean. Nature Communications, 7, 11148. 10.1038/ncomms11148 PMC4822014 27041737

[ece33325-bib-0096] Stokesbury, M. J. W. , Harvey‐Clark, C. , Gallant, J. , Block, B. A. , & Myers, R. A. (2005). Movement and environmental preferences of Greenland sharks (*Somniosus microcephalus*) electronically tagged in the St. Lawrence Estuary, Canada. Marine Biology, 148, 159–165.

[ece33325-bib-0097] Veríssimo, A. , McDowell, J. R. , & Graves, J. E. (2011). Population structure of a deep‐water squaloid shark, the Portuguese dogfish (*Centroscymnus coelolepis*). ICES Journal of Marine Science, 68, 555–563.

[ece33325-bib-0098] Vigdorchik, M. E. (1979). Isolation of the Arctic from the global ocean during glaciations. Sea Level, Ice, and Climate Change (Proceedings of the Canberra Symposium, December 1979). IAHS Publications, 131, 303–322.

[ece33325-bib-0099] Vigdorchik, M. E. , & Vinkovetsky, Y. (1976). The isolation of the Arctic basin during the glaciation as a cause of the transgression of the north of Eurasia. Annual Meeting of the Geological Society, Denver, 1153.

[ece33325-bib-0100] Walter, R. P. , Kessel, S. T. , Alhasan, N. , Fisk, A. T. , Heath, D. D. , Chekchak, T. , et al. (2014). First record of living *Manta alfredi* × *Manta birostris* hybrid. Marine Biodiversity, 44, 1–2.

[ece33325-bib-0101] Wang, J. Y. , Chou, L. S. , & White, B. N. (1999). Mitochondrial DNA analysis of sympatric morphotypes of bottlenose dolphins (genus: *Tursiops*) in Chinese waters. Molecular Ecology, 8, 1603–1612. 10583824 10.1046/j.1365-294x.1999.00741.x

[ece33325-bib-0102] Wassmann, P. , Duarte, C. M. , Agusti, S. , & Sejr, M. (2011). Footprints of climate change in the Arctic marine ecosystem. Global Change Biology, 17, 1235–1249.

[ece33325-bib-0103] Winchell, C. J. , Martin, A. P. , & Mallatt, J. (2004). Phylogeny of elasmobranchs based on LSU and SSU ribosomal RNA genes. Molecular Phylogenetics and Evolution, 31, 214–224. 15019621 10.1016/j.ympev.2003.07.010

[ece33325-bib-0104] Yano, K. , Stevens, J. D. , & Compagno, L. J. V. (2004). A review of the systematics of the sleeper shark genus *Somniosus* with redescriptions of *Somniosus* (*Somniosus*) *antarcticus* and *Somniosus* (*Rhinoscymnus) longus* (Squaliformes: Somniosidae). Ichthyological Research, 51, 360–373.

