# Supplementary material for: Origins of the Greenland shark (Somniosus microcephalus): Impacts of ice‐olation and introgression
Source: Ecol Evol. 2017 Sep 8;7(19):8113–25. doi: 10.1002/ece3.3325 (PMC5632604; doi:10.1002/ece3.3325)
Supplement: Supplementary file 2 [file ECE3-7-8113-s002.docx]

Supplementary Information

The following primers were used for both PCR amplification and sequencing - cyt *b* - *Somn-GLU-L1* GAACCATCGTTGTTTATTCAAC and *Somn-CYTB-H2* GGCAAATAGGAAATATCATTC; RAG1 - Chon-Rag1-S024a CAGATCTTCCAGCCTTTGCATC and Chon-Rag1-R022a CTGAAACCCCTTTCACTCTATC (Iglésias *et al.* 2005), - ITS2 subunit and 5.8S and 28S regions - FISH5.8SF TTAGCGGTGGATCACTCGGCTCGT and FISH28SR TCCTCCGCTTAGTAATATGCTTAAATTCAGC (Pank *et al.* 2001). All PCRs were performed in 25 μl, volumes consisting of 1x reaction buffer, 2.7 mM MgCl_2_ for cyt *b* and RAG1, 3 mM MgCl_2_ for ITS-2, 200 μM dNTPs, 0.4 μM of each primer, and 0.5 units of GenScript Taq polymerase (GenScript, USA). The thermocycler profile for the PCR reactions consisted of an initial denaturation at 95ºC for 2 min, then 30 cycles of 95ºC for 30 s, 57ºC for 30 s, 72ºC 1 min, followed by a final extension at 72ºC for 10 min, and a 4ºC soak. PCR amplicons were then sequenced in both directions using the above primers at McGill University and the Génome Québec Innovation Centre.

Table S1. Haplotypes and polymorphic positions for 702 bp DNA sequences from the mitochondrial cytochrome *b* (cyt *b*) gene used in this study. The hyphen (-) present at a nucleotide position signifies an identical nucleotide to that present in first listed haplotype (H1). *Spa = Somniosus pacificus, Smi = Somniosus microcephalus.* See attached.

Table S2 . Frequency of mitochondrial cytochrome *b* (cyt *b*) haplotypes study-wide. AK = Alaska, CS = Cumberland Sound, DI = Disko Island, GF = Grice Fjord, IC = Iceland, KP = Kakiak Point, MB = Maxwell Bay, NS = Nova Scotia, RB = Resolute Bay, SI = Scott Inlet, SV = Svalbard, GM = Gulf of Mexico, MA = Mid-Atlantic Ridge, GB = GenBank. See attached Excel sheet. *Spa = Somniosus pacificus, Smi = Somniosus microcephalus* n = sample size, S = number of polymorphic sites, h = number of haplotypes, Hd = haplotype diversity, k = average number of sequence pairwise differences, π = nucleotide diversity. See attached.
